# Treatment of OPG-deficient mice with WP9QY, a RANKL-binding peptide, recovers alveolar bone loss by suppressing osteoclastogenesis and enhancing osteoblastogenesis

**DOI:** 10.1371/journal.pone.0184904

**Published:** 2017-09-22

**Authors:** Yuki Ozaki, Masanori Koide, Yuriko Furuya, Tadashi Ninomiya, Hisataka Yasuda, Midori Nakamura, Yasuhiro Kobayashi, Naoyuki Takahashi, Nobuo Yoshinari, Nobuyuki Udagawa

**Affiliations:** 1 Graduate School of Oral Medicine, Matsumoto Dental University, Shiojiri, Nagano, Japan; 2 Institute for Oral Science, Matsumoto Dental University, Shiojiri, Nagano, Japan; 3 Nagahama Institute for Biochemical Science, Oriental Yeast Co., Ltd., Shiga, Japan; 4 Department of Biochemistry, Matsumoto Dental University, Shiojiri, Nagano, Japan; 5 Department of Periodontology, Matsumoto Dental University, Shiojiri, Nagano, Japan; Universite de Nantes, FRANCE

## Abstract

Osteoblasts express two key molecules for osteoclast differentiation, receptor activator of NF-κB ligand (RANKL) and osteoprotegerin (OPG), a soluble decoy receptor for RANKL. RANKL induces osteoclastogenesis, while OPG inhibits it by blocking the binding of RANKL to RANK, a cellular receptor of RANKL. *OPG*-deficient (*OPG*^–/–^) mice exhibit severe alveolar bone loss with enhanced bone resorption. WP9QY (W9) peptide binds to RANKL and blocks RANKL-induced osteoclastogenesis. W9 is also reported to stimulate bone formation *in vivo*. Here, we show that treatment with W9 restores alveolar bone loss in *OPG*^–/–^mice by suppressing osteoclastogenesis and enhancing osteoblastogenesis. Administration of W9 or risedronate, a bisphosphonate, to *OPG*^–/–^mice significantly decreased the osteoclast number in the alveolar bone. Interestingly, treatment with W9, but not risedronate, enhanced Wnt/β-catenin signaling and induced alveolar bone formation in *OPG*^–/–^mice. Expression of sclerostin, an inhibitor of Wnt/β-catenin signaling, was significantly lower in tibiae of *OPG*^–/–^mice than in wild-type mice. Treatment with risedronate recovered sclerostin expression in *OPG*^–/–^mice, while W9 treatment further suppressed sclerostin expression. Histomorphometric analysis confirmed that bone formation-related parameters in *OPG*^–/–^mice, such as osteoblast number, osteoblast surface and osteoid surface, were increased by W9 administration but not by risedronate administration. These results suggest that treatment of *OPG*^–/–^mice with W9 suppressed osteoclastogenesis by inhibiting RANKL signaling and enhanced osteoblastogenesis by attenuating sclerostin expression in the alveolar bone. Taken together, W9 may be a useful drug to prevent alveolar bone loss in periodontitis.

## Introduction

Osteoclasts, bone-resorbing multinucleated cells, differentiate from the monocyte/macrophage lineage under the tight control of osteoblast-lineage cells (hereafter called osteoblasts) [1, 2]. Osteoblasts express two key molecules for osteoclast differentiation, receptor activator of NF-κB ligand (RANKL) [3, 4] and osteoprotegerin (OPG) [5, 6]. RANKL is a member of the tumor necrosis factor (TNF) superfamily that binds to its receptor RANK expressed on osteoclasts and their progenitors [1, 2]. The interaction of RANKL with RANK is required for osteoclast differentiation and activation. OPG, a soluble decoy receptor for RANKL, inhibits osteoclastogenesis by blocking the RANKL-RANK interaction. *OPG*-deficient (*OPG*^–/–^) mice exhibit severe osteopenia with enhanced bone resorption [7, 8]. Bone formation of *OPG*^–/–^mice is also enhanced through the coupling mechanism [9]. We and others have reported that *OPG*^–/–^mice demonstrate severe alveolar bone loss [10, 11].

Alveolar bone, a component of periodontal tissues, supports teeth and protects them from biting forces. Periodontitis is an inflammatory disease characterized by destruction of periodontal tissues including alveolar bone [12]. Bacterium-derived factors, such as lipopolysaccharide (LPS) and antigens, activate the innate and acquired immune system, respectively [13]. Activation of both systems induces local inflammatory reactions. Eventually, a cascade of inflammatory reactions leads to osteoclastogenesis and subsequent progression of bone loss, which in turn causes the loss of teeth.

Treatments of humans and animals with antiresorptive agents, such as bisphosphonates and denosumab, an anti-RANKL neutralizing monoclonal antibody, have been shown to inhibit bone resorption associated with osteoporosis and periodontitis [11, 14–16]. Administration of bisphosphonates significantly prevented alveolar bone loss in a rat periodontitis model [14]. It was also reported that osteoporotic patients treated with bisphosphonates tended to have fewer missing teeth than untreated [15, 16]. We reported that treatment of *OPG*^–/–^mice with risedronate, a bisphosphonate, or anti-RANKL antibody significantly reduced the osteoclast number in the alveolar bone and protected the alveolar bone loss [11]. These findings indicate that the *OPG*^–/–^mouse is a useful model for screening antiresorptive agents.

Bone resorption is reported to be tightly coupled with bone formation [17–20]. This coupling phenomenon is clearly observed in *OPG*^–/–^mice [7, 9]. Bone formation in *OPG*^–/–^mice is accelerated concomitantly by the increased bone resorption. Treatment of *OPG*^–/–^mice with risedronate, and that of wild-type (WT) mice with anti-RANKL antibodies inhibited not only bone resorption but also bone formation [9, 20]. Several factors are proposed as coupling factors that facilitate the transition from bone resorption to bone formation. Sphingosine-1-phosphate (S1P) secreted from osteoclasts promotes osteoblast differentiation [21]. It is also proposed that some factors embedded in bone matrix, such as transforming growth factor β and bone morphogenetic proteins, are released as coupling factors during osteoclastic bone resorption [22]. Wnt/β-catenin signaling in osteoblasts promotes bone formation *in vivo* [23]. We have proposed that osteocytes contribute to the coupling process through the regulation of expression of sclerostin (encoded by the *Sost* gene), an antagonist of Wnt/β-catenin signaling [24]. Expression of sclerostin was markedly suppressed in *OPG*^–/–^mice [25], and administration of anti-RANKL antibodies to *OPG*^–/–^mice enhanced sclerostin expression [24]. Administration of bisphosphonates to mice prevented ovariectomy-induced bone loss, and also enhanced sclerostin expression [26]. These results suggest that osteoclastic bone resorption may regulate sclerostin expression by osteocytes.

WP9QY (W9) is a peptide designed to be structurally similar to one of the cysteine-rich domains in TNF receptor type I [27]. W9 was demonstrated to bind not only TNF-α but also RANKL, and to inhibit RANKL-induced osteoclastogenesis *in vitro* [28]. Administration of W9 increased the bone mineral density (BMD) through inhibition of osteoclastogenesis in several mouse bone-loss models. In addition, W9 stimulated osteoblast differentiation in osteoblast cultures in part through the production of BMP-2 [29]. Therefore, it is proposed that W9 binds RANKL expressed on osteoblasts and induces RANKL-mediated reverse signals in osteoblasts to induce BMP-2 production. Local administration of W9 also augmented BMP-2-induced ectopic bone formation in mice [30]. These reports suggest that W9 administration increases BMD through both inhibition of bone resorption and induction of bone formation.

Here, we examined the effects of W9 administration on alveolar bone loss in *OPG*^–/–^mice. Treatment of *OPG*^–/–^mice with W9 suppressed osteoclastogenesis by inhibiting RANKL signaling and enhanced osteoblastogenesis by attenuating sclerostin expression in alveolar bone. Thus, we propose that W9 may be a useful drug to prevent alveolar bone loss in periodontitis.

## Materials and methods

### Mice and reagents

*OPG*^–/–^mice (genetic background C57BL/6) [8] were purchased from CLEA Japan (Tokyo, Japan). *OPG*^–/–^mice were bred with WT mice (strain C57BL/6) obtained from Japan SLC (Shizuoka, Japan). Twelve-week-old male *OPG*^–/–^ and *OPG*^*+/+*^ littermates (WT) were used in this study. The number of mice in the respective group was determined according to our previous studies [11, 24]. All procedures for animal care were approved by the Animal Management Committee of Matsumoto Dental University (permit number: 267) and performed accordingly. W9 peptide [29] was provided by Oriental Yeast (Tokyo). Risedronate was obtained from LKT laboratories (St. Paul, MN). Other chemicals and reagents were of analytical grade.

### Treatments of *OPG*^–/–^ and WT mice with W9 and risedronate

**Fig 1:**
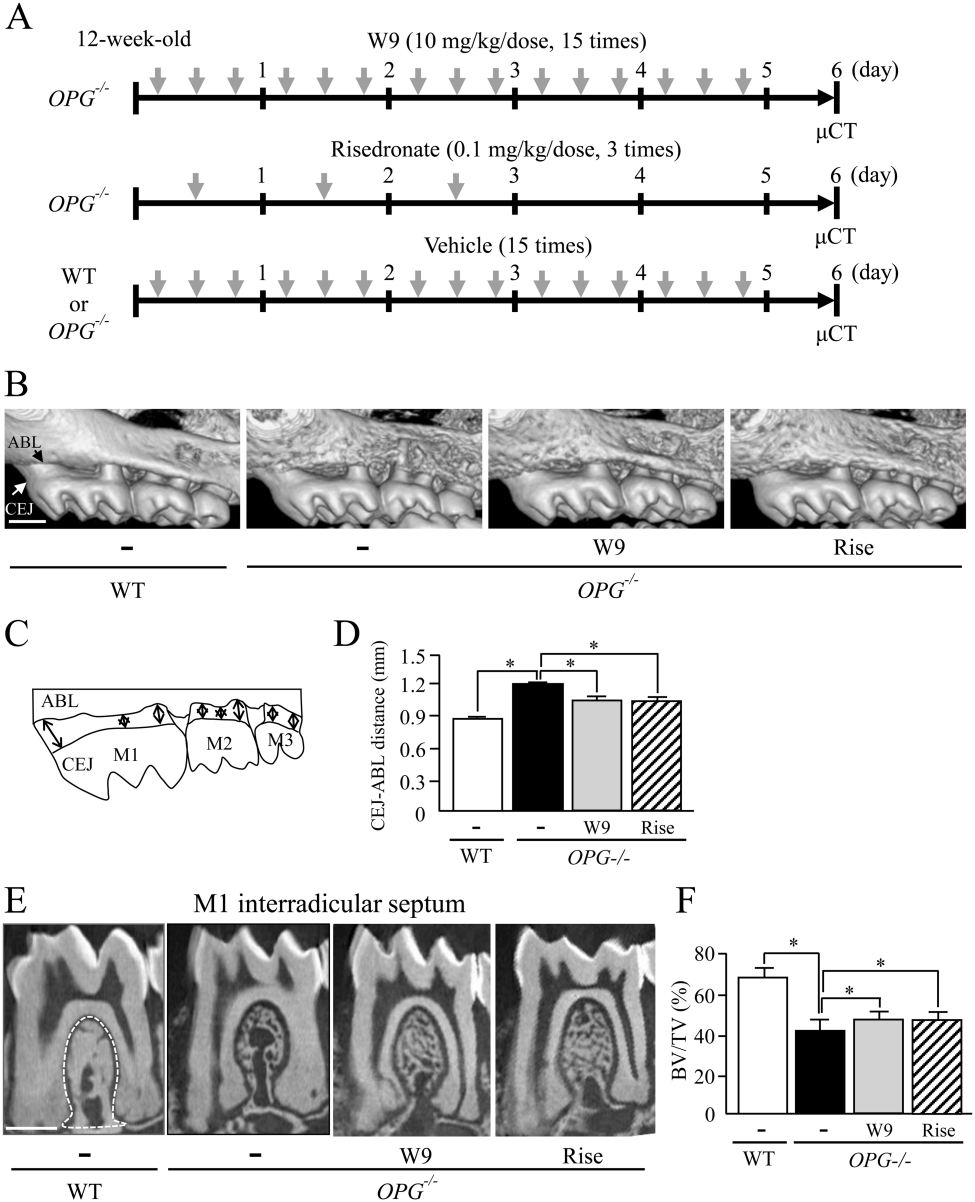
Effects of W9 and risedronate administration on alveolar bone loss in *OPG*^*-/-*^ mice. (A) Experimental design. W9 or vehicle was administered subcutaneously three times/day for the first 5 days to 12-week-old male *OPG*^–/–^mice. Risedronate was administered once a day at 0.1 mg/kg body weight for the first 3 days. Saline (vehicle) was administered to *OPG*^–/–^ and WT mice similarly to W9 administration. Each group of mice was sacrificed on day 6 (*n* = 7). (B) Three-dimensional μCT images of maxillae from WT mice and *OPG*^–/–^mice. (C) A schematic diagram for measurement of the distance between CEJ and ABC on μCT images. (D) The CEJ-ABC distance in WT mice and in *OPG*^–/–^mice. (E) μCT images of the interradicular septum of the first molar (M1) in mandibles from WT mice (an area surrounded by a white dotted line) and *OPG*^–/–^mice. (F) Bone volume/tissue volume (BV/TV) was measured in the M1 interradicular septum from WT mice and in *OPG*^–/–^mice treated with or without W9 and risedronate (*n* = 7). Data are expressed as the mean ± SD in (D) and (F). *: p<0.05. Scale bar, 0.5 mm.

W9 or risedronate, a nitrogen-containing bisphosphonate, was subcutaneously administered to 12-week-old *OPG*^–/–^mice (*n* = 7 for each group) (Fig 1A). W9 was administered three times a day at 10 mg/kg/dose body weight for the first 5 days [29]. Risedronate was administered once a day at 0.1 mg/kg body weight for the first 3 days. Saline (vehicle) was administered to 12-week-old *OPG*^–/–^ and WT mice similarly to W9 administration. Calcein (a fluorescent dye for labeling of bones) was injected twice subcutaneously into mice on day 1 and day 4. Mice were sacrificed on day 6 by injection of excess amounts of pentobarbital.

### Analysis of alveolar bone loss by *in vivo* μCT images

Maxillae of WT and *OPG*^–/–^mice (*n* = 7) were analyzed on day 6 using an *in vivo* μCT apparatus (R_mCT; Rigaku, Tokyo) at 85 kV and 160 μA with a copper filter of 0.1 mm using 512 projections over 30 s [11]. The voxel resolution was 20 μm. The images reconstructed were observed using i-view software (J. Morita Mfg., Kyoto, Japan). The distance between the cemento–enamel junction (CEJ) and alveolar bone crest (ABC) was measured at 8 points for each molar [first molar (M1) to third molar (M3)] of maxillae as alveolar bone loss, a clinical parameter in periodontitis (Fig 1C). The distance of 8 points was summed as alveolar bone loss.

### Bone morphometry of interradicular septum in first molars

Mandibles were subjected to the three-dimensional (3D) μCT analysis using micro focus X-ray CT (ScanXmate-A080; Comscantecno, Yokohama, Japan). A sagittal surface that penetrated the medial and distal root canals of the M1 of mandibles was used for observation. The region of interest (ROI) was manually established in the alveolar bone area of the M1 interradicular septum (Fig 1E, left panel). The inferior border of the ROI was determined by drawing a line between the root apexes of the medial and distal roots of the M1 [11]. The CT images were used to reconstruct 3D images. Bone morphometric analysis to determine BV/TV (*n* = 7) was performed using analytic software (TRI/3D-BON; Ratoc System Engineering, Tokyo).

### Histomorphometry of alveolar bone

**Fig 2:**
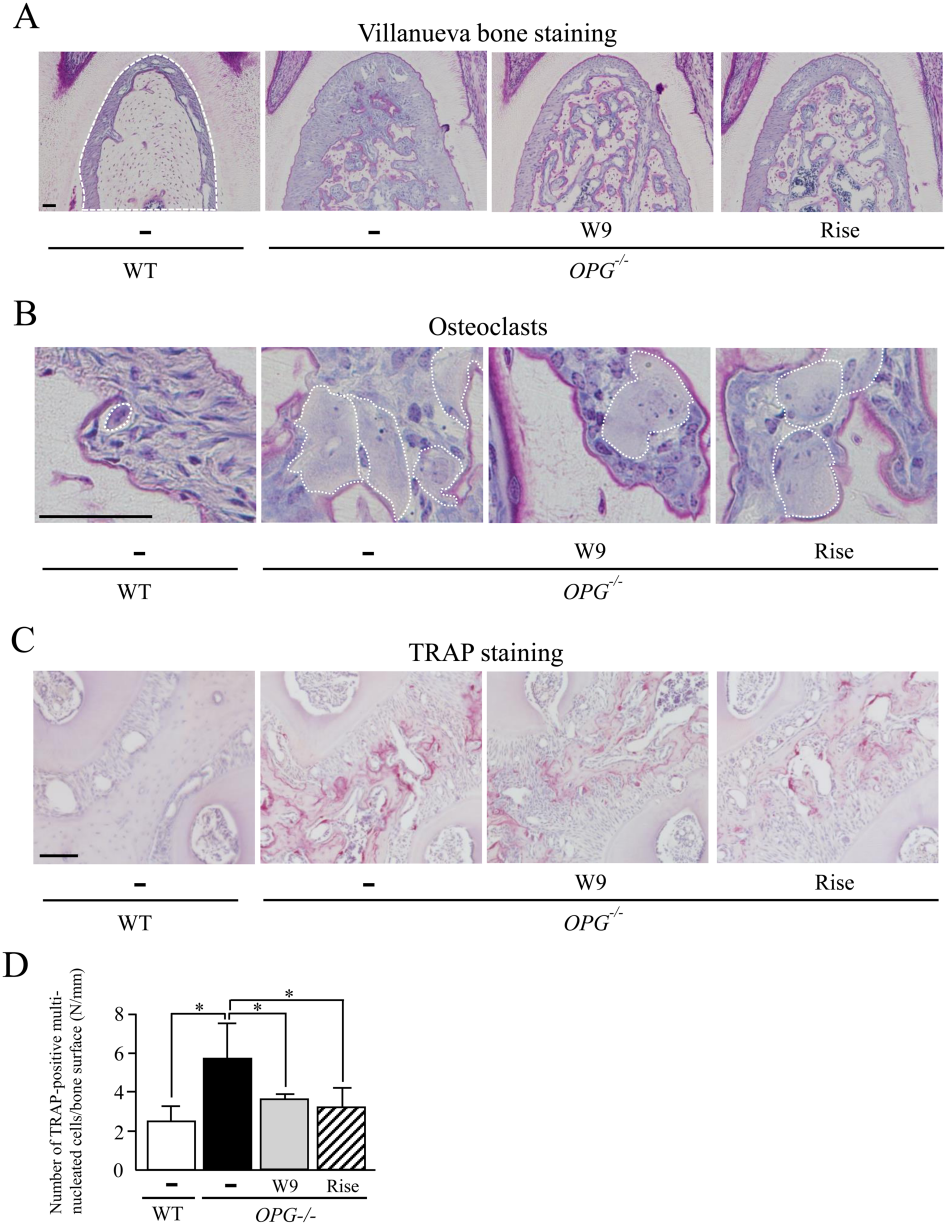
Effects of W9 and risedronate administration on alveolar bone resorption in *OPG*^*-/-*^ mice. (A) Histological analysis of the interradicular septum of the first molar (M1) in mandibles from WT mice and *OPG*^–/–^mice treated with and without W9 or risedronate. Mandible tissues were subjected to Villanueva bone staining. (B) Osteoclasts in the M1 interradicular septum from WT and *OPG*^–/–^mice. Multinucleated osteoclasts are surrounded by white dotted lines. (C) TRAP staining of maxillae from WT and of *OPG*^–/–^mice. TRAP-positive osteoclasts (red cells) were observed in the M1 interradicular septum in alveolar bone areas. (D) The number of TRAP-positive cells/bone surface (N/mm) was determined in the M1 interradicular septum (*n* = 5). Data are expressed as the means ± SD. *: p<0.05. Scale bar, 50 μm.

To examine bone resorption and formation in the alveolar bone, the mandibles were recovered from WT and *OPG*^–/–^mice. Mandibles were fixed in 70% ethanol and embedded in glycol-methacrylate without decalcification. Sections were prepared and stained with Villanueva Goldner to discriminate between mineralized and unmineralized bone, and to identify cellular components. The ROI was manually established in the alveolar bone area of the M1 interradicular septum (Fig 2A, left panel). Quantitative histomorphometric analysis was performed in a blind fashion (*n* = 5). Nomenclature and units were used according to the guidelines of the histomorphometry nomenclature committee of the American Society for Bone and Mineral Research [31].

### Immunocytochemistry of alveolar bone

The dissected maxillae were used for immunohistological analysis. They were fixed in 4% paraformaldehyde (PFA), decalcified with 10% EDTA, and embedded in paraffin. Histological sections were prepared and stained for TRAP, a marker of osteoclasts. For immunohistochemical staining, histological sections were incubated with the anti-osterix antibody (ab22552, abcam, Cambridge, UK), anti-ALP antibody (ab108337, abcam), anti-β-catenin antibody (ab32572, abcam), or anti-sclerostin antibody (AF1589, R&D Systems, Minneapolis, MN) at 4°C overnight. After washing, the sections were incubated with the horseradish peroxidase (HRP)-conjugated secondary antibody. The HRP-conjugated antibody was visualized with a 3,3'-diaminobenzidine tetrahydrochloride (DAB) kit (DAKO, Carpinteria, CA). The sections were counterstained with hematoxylin. The number of TRAP- or osterix-, sclerostin-positive cells was measured by histomorphometry, in the alveolar bone of maxillae, and expressed as cells/bone surface or cells/bone area. The surface and area of bone in the alveolar bone of maxillae were measured by ImageJ software (NIH, Bethesda, MD, USA; http://rsb.info.nih.gov/ij).

### Measurements of a serum marker

Mouse serum samples were collected and subjected to ELISA analysis as described previously [11]. Serum activities of ALP were determined with an ALP assay kit (Wako, Osaka, Japan).

### Statistical analysis

The experiments of measuring of distances between CEJ and ABL using μCT images and bone volume of M1 interradicular septum were repeated twice and similar results were obtained. The results are expressed as the mean ± SD for five or more animals. The significance of differences between two groups was determined by Student’s *t*-test and that among 3 or more groups was determined by ANOVA with a Fisher’s protected least significant difference test.

## Results

### W9 restores alveolar bone loss in *OPG*^–/–^mice

We first examined whether administration of W9 suppresses the alveolar bone loss in *OPG*^–/–^mice using μCT (Fig 1). W9 or risedronate was injected into 12-week-old *OPG*^–/–^mice or WT mice, and alveolar bone loss was evaluated on day 6 (Fig 1A). The alveolar bone surface in *OPG*^–/–^mice was much rougher than that in WT mice (Fig 1B). *OPG*^–/–^mice also exhibited severe alveolar bone resorption associated with exposed dental roots of molars. Administration of W9 or risedronate to *OPG*^–/–^mice markedly reduced the cortical porosity on day 6 (Fig 1B). The distance between the cemento–enamel junction (CEJ) and alveolar bone crest (ABC) (CEJ-ABC distance), used as an indicator of alveolar bone loss, was determined on 3D images of maxillae (Fig 1C). The CEJ-ABC distance in *OPG*^–/–^mice was significantly greater than that in WT mice (Fig 1D). Administration of W9 or risedronate to *OPG*^–/–^mice significantly reduced the CEJ-ABC distance compared with vehicle-treated *OPG*^–/–^mice. To further evaluate the resorption of alveolar bone in *OPG*^–/–^mice, we measured bone volume of the M1 interradicular septum, a part of alveolar bone. μCT analysis revealed that bone volume/tissue volume (BV/TV) in the septum was significantly lower in *OPG*^–/–^mice than in WT mice (Fig 1E and 1F). Administration of W9 or risedronate significantly increased BV/TV of this region in *OPG*^–/–^mice, but not in WT mice (Fig 1F and S3 Fig). These results suggest that the alveolar bone loss in *OPG*^–/–^mice is restored by administration of W9 or risedronate.

### W9 suppresses osteoclastogenesis in alveolar bone of *OPG*^–/–^mice

We next examined bone tissues of the M1 interradicular septum using histomorphometric analysis (Fig 2). Villanueva bone staining revealed that osteocytes of the M1 interradicular septum were arranged the layered structures (Fig 2A, left panel), indicating that the interradicular septum was developed by lamella bone formation. Bone area/tissue area (B.Ar/T.Ar) of the septum was significantly lower in *OPG*^–/–^mice than in WT mice (Fig 2A, Table 1). Administration of W9 or risedronate to *OPG*^–/–^mice significantly increased B.Ar/T.Ar of the M1 interradicular septum.

**Table 1:** Histomorphometric analysis of the protective effects of W9 on alveolar bone loss in *OPG*^*-/-*^ mice.

Parameter	-	-	W9	Risedronate
	WT	OPG^-/-^	OPG^-/-^	OPG^-/-^
B.Ar/T.Ar (%)	54.2±6.7	25.3±4.0^a^	37.1±3.1^a^^,^ ^b^	32.6±2.9^a^^,^ ^b^
N.OC/BS (N/mm)	0.6±0.3	6.8±2.6^a^	2.4±0.6^b^	2.6±0.6^a^^,^ ^b^
OC S/BS (%)	2.4±1.2	21.5±8.1^a^	9.4±3.2^a^^,^ ^b^	7.3±2.2^b^
N.OB/BS (N/mm)	19.0±4.7	25.4±5.0	49.4±6.4^a^^,^ ^b^	29.8±5.7^a^^,^ ^c^
OB S/BS (%)	24.4±6.2	25.8±6.2	52.0±4.7^a^^,^ ^b^	31.7±6.8^c^
MAR (μm/day)	2.0±0.3	3.8±0.7^a^	3.0±0.4^a^^,^ ^b^	3.1±0.4^a^^,^ ^b^
BFR/BS (mm^3^/mm^2^/year)	0.32±0.09	0.43±0.11^a^	0.45±0.05^a^	0.40±0.07
OS/BS (%)	31.6±7.4	41.3±7.6	69.6±5.8^a^^,^ ^b^	47.2±8.9^a^^,^ ^c^
Mlt. (day)	1.10±0.22	0.89±0.19	2.15±0.15^a^^,^ ^b^	1.34±0.25^b^^,^ ^c^

^a)^ *P* < 0.05 versus vehicle treated WT group,

^b)^ *P* < 0.05 versus vehicle treated *OPG*^*-/-*^ group,

^c)^ *P* < 0.05 versus W9 treated *OPG*^*-/-*^ group.

Bone area, B.Ar; Tissue area, T.Ar; Osteoclast number, N.Oc; Bone surface, BS; Osteoclast surface, Oc.S; Osteoblast number, N.Ob; Osteoblast surface, Ob.S; Mineral apposition rate, MAR; Bone formation rate, BFR; Osteoid surface, OS; Mineralization lag time, Mlt.

Osteoclastogenesis was also examined in the M1 interradicular septum, in which osteoclasts were hardly observed in WT mice (Fig 2B, left panel). Osteoclasts were indicated by white dotted lines. The osteoclast number was significantly higher in *OPG*^–/–^mice than in WT mice (Fig 2B, Table 1). The size of osteoclasts in *OPG*^–/–^mice was larger than that in WT mice (Fig 2B). Administration of W9 and risedronate to *OPG*^–/–^mice significantly decreased the osteoclast number compared with vehicle administration, without apparent effects on the size of osteoclasts (Fig 2B, Table 1). Osteoclastogenesis in the M1 interradicular septum was also evaluated by tartrate-resistant acid phosphatase (TRAP, a marker of osteoclasts) staining (Fig 2C). TRAP-positive osteoclasts and cement lines were more abundant in *OPG*^–/–^mice than in WT mice, suggesting that both osteoclast formation and osteoclast function in the septum were accelerated in *OPG*^–/–^mice. Administration of W9 and risedronate significantly decreased the number of TRAP-positive osteoclasts in this region (Fig 2D). The inhibitory effects of W9 on osteoclastogenesis were milder than those of risedronate. These results suggest that the increased number of osteoclasts in the M1 interradicular septum of *OPG*^–/–^mice is suppressed by administration of W9 and risedronate.

### W9 enhances osteoblastogenesis in alveolar bone of *OPG*^–/–^mice

**Fig 3:**
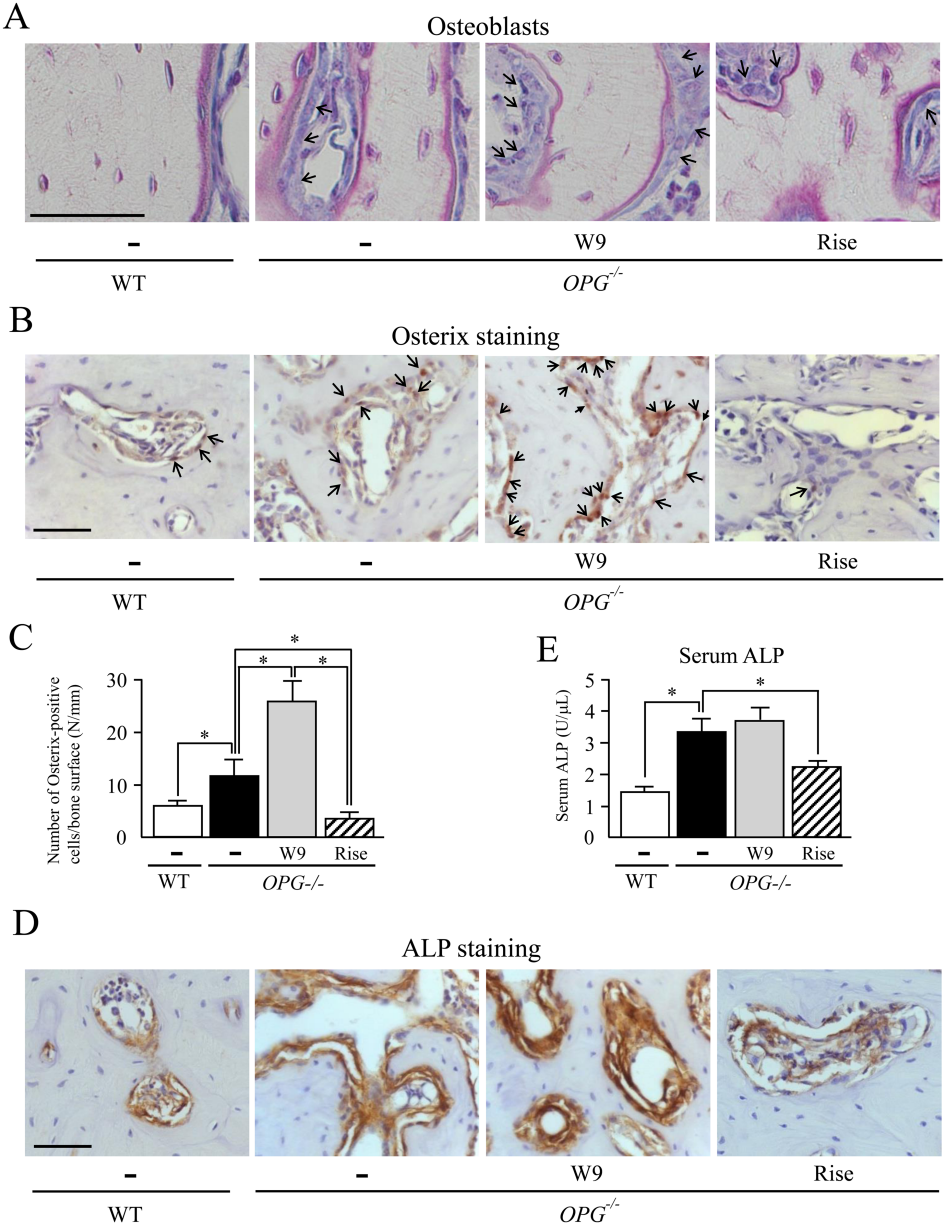
Effects of W9 and risedronate administration on alveolar bone formation in *OPG*^*-/-*^ mice. (A) Histological analysis of the interradicular septum of the first molar (M1) in mandibles from WT and *OPG*^–/–^mice treated with and without W9 or risedronate. Mandible tissues were subjected to Villanueva bone staining. Mature osteoblasts were indicated by arrows. (B) Osterix staining of maxillae from WT and *OPG*^–/–^mice. Osterix-positive cells in nuclei (brown, arrows) were observed in the M1 interradicular septum in alveolar bone areas. (C) The number of osterix-positive cells/bone surface (N/mm) was determined in the M1 interradicular septum (*n* = 5). (D) ALP staining of maxillae from WT and *OPG*^–/–^mice. ALP-positive cells were observed in the M1 interradicular septum in alveolar bone areas. (E) Serum ALP activities were measured with an ALP kit (*n* = 7). Data are expressed as the mean ± SD in (C) and (E). *: p<0.05. Scale bar, 50 μm.

To elucidate effects of W9 on alveolar bone formation in *OPG*^–/–^mice, we evaluated the osteoblast number in the M1 interradicular septum (Fig 3A). The flat cells, identified as inactivated osteoblasts, were frequently observed in WT mice. Activated osteoblasts were identified as cuboidal cells on bone surfaces in this region (black arrows) in *OPG*^–/–^mice. The osteoblast number in the septum tended to be higher in *OPG*^–/–^mice than in WT mice (Fig 3A, Table 1). The thickness of osteoblasts was also increased by approximately 2-fold in *OPG*^–/–^mice, compared with WT mice. Administration of W9 to *OPG*^–/–^mice significantly increased the osteoblast number (Fig 3A, Table 1). The thickness of osteoblasts appeared to be increased by W9 administration (Fig 3A). In contrast, administration of risedronate to *OPG*^–/–^mice failed to affect osteoblast number in the M1 interradicular septum (Table 1). Both MAR and bone formation rate (BFR) were significantly higher in *OPG*^–/–^mice than WT mice (Table 1). Administration of W9, but not risedronate, significantly increased both osteoblast surface and osteoid surface (Table 1). These results suggest that bone formation in the M1 interradicular septum is further accelerated in *OPG*^–/–^mice treated with W9.

Osterix is a master transcription factor for osteoblast differentiation expressed in osteogenic cells [32]. We further examined bone formation in the M1 interradicular septum by immunostaining of osterix. The number of osterix-positive osteoblasts was significantly higher in *OPG*^–/–^mice than in WT mice (Fig 3B and 3C). Administration of W9 to *OPG*^–/–^mice significantly increased the number of the osterix-positive osteoblasts. In contrast, administration of risedronate to *OPG*^–/–^mice significantly decreased the osterix-positive osteoblast number (Fig 3B and 3C). Immunostaining of ALP, a marker of osteoblasts, showed that expression of ALP in the M1 interradicular septum was much stronger in *OPG*^–/–^mice than in WT mice (Fig 3D). Administration of risedronate to *OPG*^–/–^mice suppressed ALP expression, suggesting that osteoblast function was suppressed by treatment with risedronate. However, W9 administration failed to decrease ALP expression in osteoblasts in *OPG*^–/–^mice (Fig 3D). These findings were supported by measurement of serum levels of ALP. The serum level of ALP was higher in *OPG*^–/–^mice than in WT mice (Fig 3E). Administration of risedronate significantly decreased the serum level of ALP in *OPG*^–/–^mice, while W9 did not (Fig 3E). These results suggest that osteoblast differentiation in the M1 interradicular septum is accelerated in *OPG*^–/–^mice, and that administration of W9, but not risedronate, to *OPG*^–/–^mice enhances osteoblast differentiation in this region.

### W9 enhances Wnt/β-catenin signaling of alveolar bone in *OPG*^–/–^mice

**Fig 4:**
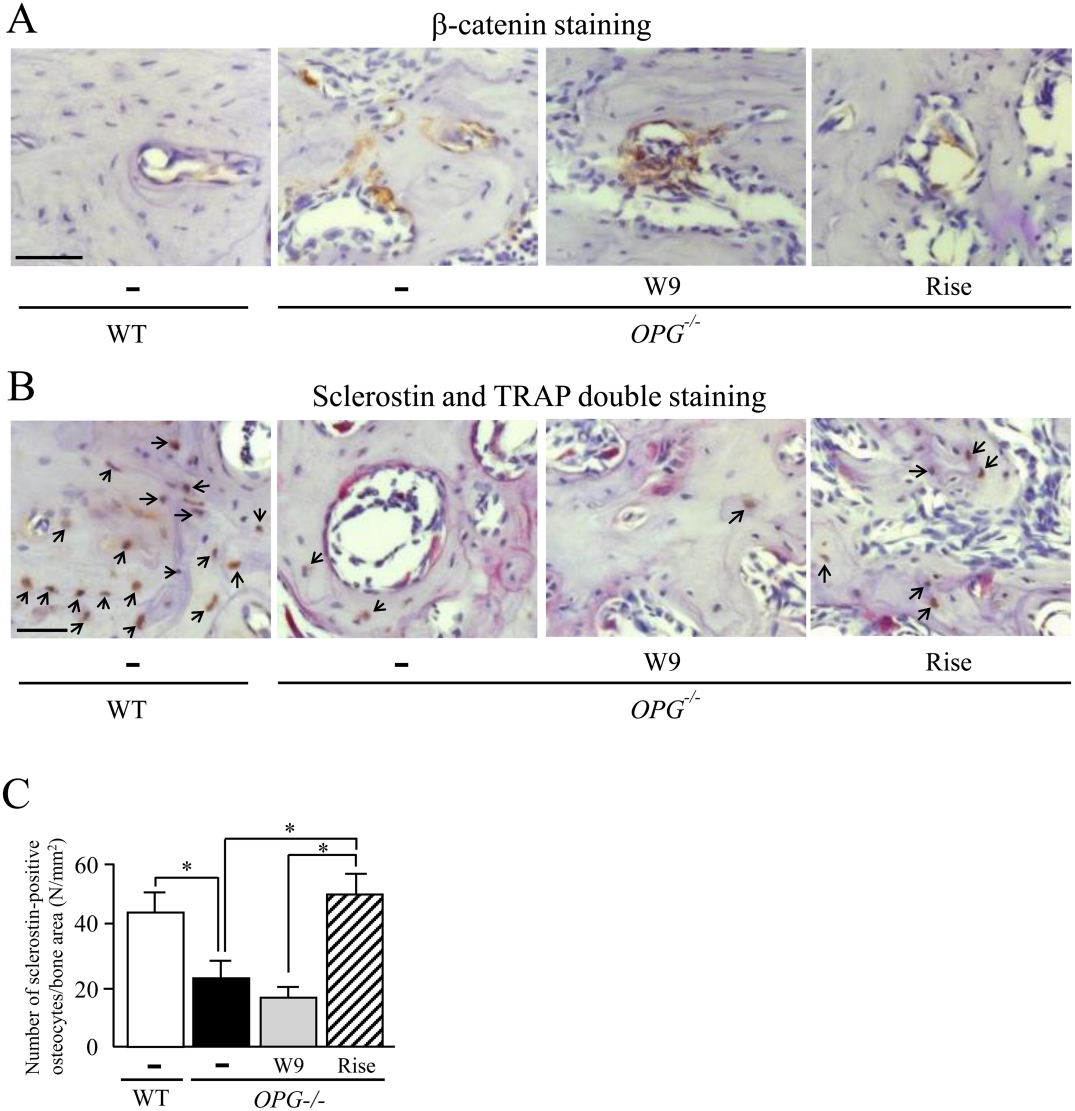
Effects of W9 and risedronate administration on Wnt/β-catenin signaling of alveolar bone in *OPG*^*-/-*^ mice. (A) Histological analysis of the interradicular septum of the first molar (M1) in maxillae from WT and *OPG*^–/–^mice treated with and without W9 or risedronate. β-catenin staining of WT and *OPG*^–/–^mice. β-catenin-positive cells in nuclei (brown) were observed in the M1 interradicular septum in alveolar bone areas. (B) Sclerostin and TRAP double staining of WT and *OPG*^–/–^mice. Sclerostin-positive osteocytes (brown) were observed in the M1 interradicular septum in alveolar bone areas. Sclerostin-positive osteocytes are indicated by black arrows. (C) The number of sclerostin-positive cells/bone area (N/mm^2^) was determined in the M1 interradicular septum (*n* = 5). Data are expressed as the mean ± SD. *: p<0.05. Scale bar, 50 μm.

To clarify the functional mechanisms of W9 on bone formation, we next examined the expression of β-catenin in alveolar bone in *OPG*^–/–^mice treated with W9 or risedronate, using immunostaining with anti-β-catenin antibodies. β-catenin-positive signals on bone surfaces in the M1 interradicular septum were stronger in *OPG*^–/–^mice than in WT mice (Fig 4A). Administration of W9 to *OPG*^–/–^mice enhanced β-catenin-positive signals (Fig 4A). In contrast, administration of risedronate attenuated β-catenin-positive signals. These results suggest that Wnt/β-catenin signaling is increased in *OPG*^–/–^mice, and W9 administration further enhances Wnt/β-catenin signals in osteoblasts.

It was reported that the expression of sclerostin in osteocytes is lower in *OPG*^–/–^mice [25]. We finally examined whether the treatment with W9 affects the expression of sclerostin in alveolar bone in *OPG*^–/–^mice using both immunostaining of sclerostin and histochemical staining of TRAP. Administration of W9 to *OPG*^–/–^mice decreased the number of osteoclasts (Fig 4B, also see Fig 2D). However, the sclerostin signal in osteocytes was rather suppressed by W9 administration (Fig 4B and 4C). Administration of risedronate suppressed the osteoclast number and enhanced sclerostin signals in osteocytes in *OPG*^–/–^mice (Fig 4B and 4C). Thus, administration of W9, but not risedronate, to *OPG*^–/–^mice enhanced Wnt/β-catenin signaling in alveolar bone through attenuation of sclerostin expression. These results suggest that W9-induced suppression of sclerostin expression by osteocytes is independent of W9-induced inhibition of osteoclastogenesis.

## Discussion

In the present study, we demonstrated that treatment of *OPG*^–/–^mice with W9 restored their alveolar bone loss through two independent mechanisms: One is suppression of osteoclastogenesis by inhibiting RANK signaling in osteoclast precursors, and the other is enhancement of osteoblastogenesis by suppressing sclerostin expression in osteocytes. In addition to W9, OP3-4, a cyclic peptide, to mimic OPG, was reported to bind to RANKL, thereby inhibiting osteoclastogenesis [33]. OP3-4 could accelerate bone formation by affecting RANKL signaling in osteoblasts in a similar manner to W9 [33]. These results suggest that RANKL-binding peptides may have unique characteristics in the regulation of bone resorption and formation.

*OPG*^–/–^mice exhibited a high rate of bone turnover, in which bone formation was accelerated with enhanced bone resorption [7, 9]. We have proposed that the primary cause of the coupling phenomenon in *OPG*^–/–^mice is the regulation of sclerostin expression by osteocytes. Sclerostin expression was maintained low in *OPG*^–/–^mice [24, 25]. When an anti-RANKL antibody was administered to *OPG*^–/–^mice, sclerostin expression in osteocytes was enhanced concomitantly with the suppression of osteoclastic bone resorption [24]. We have also shown that osteoclasts secreted a factor which inhibited the expression of sclerostin in UMR 106 cells, an osteoblast lineage cell line [24]. Present study showed that risedronate administration to *OPG*^–/–^mice restored the expression level of sclerostin in osteocytes to that of WT mice. In addition, clinical studies reported that bone resorption contributed to the bone turnover state [18, 19] and was associated with the serum level of sclerostin [34–36]. Treatment with bisphosphonate suppressed bone turnover and increased serum sclerostin levels in postmenopausal women [36]. These results suggest that the regulatory expression of sclerostin by osteocytes plays a role in the coupling between bone resorption and formation in *OPG*^–/–^mice.

Administration of W9 as well as risedronate to *OPG*^–/–^mice suppressed bone resorption. W9 is reported to inhibit the RANKL-RANK interaction, similar to OPG. Indeed, W9 suppressed osteoclast formation in bone marrow macrophage cultures treated with RANKL [28, 29]. W9 also inhibited survival of osteoclasts supported by RANKL but not by interleukin (IL) **-**1α (S1 Fig). W9 has been shown to get into a specific pocket in the RANKL-RANK binding structure [28]. Thus, the inhibitory effects of W9 on osteoclastic bone resorption in *OPG*^–/–^mice may be due to the inhibition of the RANKL-RANK interaction in osteoclast precursors.

In sharp contrast to risedronate, W9 administration did not suppress bone formation, but keep low levels of sclerostin expression in *OPG*^–/–^mice. W9 added to UMR106 cell cultures suppressed the secretion of sclerostin (data not shown). These results suggest that W9 enhances bone formation through the suppression of sclerostin expression in osteocytes. W9 was also reported to enhance the ectopic bone formation induced by BMP-2 [30]. BMP-2-induced osteoblastic differentiation was up-regulated by W9 in C2C12 cell cultures. We examined the effects of W9 on osteoblastic differentiation in *OPG*^–/–^calvarial osteoblasts *in vitro* (S2 Fig). The intensity of W9-induced ALP activity and mineralization in *OPG*^–/–^calvarial osteoblast cultures were equivalent to those in WT cultures. These results indicated that W9 induced osteoblast differentiation independently of OPG signaling. These results suggest that W9 directly acts on osteoblasts and osteocytes to enhance bone formation.

It is proposed that W9 and OP3-4 bind to RANKL expressed on cell surfaces of osteoblasts to induce retrograde signals [29, 33]. Up-regulation of the alkaline phosphatase expression by these peptides was not clearly observed in osteoblasts derived from *RANKL*^*–/–*^mice *in vitro* [29]. W9 and OP3-4 have been demonstrated to stimulate mammalian target of rapamycin complex 1 (mTORC1) signaling in osteoblasts, which may be necessary for the RANKL-mediated retrograde signals [33]. It was also reported that RANK-coated beads enhanced OPG secretion by osteoblasts through the interaction between RANK expressed on beads and RANKL expressed on osteoblasts [37]. These results suggest that W9 as well as RANK may induce RANKL-mediated retrograde signals in osteoblasts to increase bone mass. OPG, which most effectively binds to RANKL with very high affinity, did not exhibit such a reverse effect on osteoblasts, suggesting that the binding formula of OP3-4 and W9 to RANKL may be different from that of OPG. Further experiments are necessary to elucidate the precise mechanism of action of W9 in bone formation.

Administration of W9 to 12-week-old WT mice failed to increase BV/TV in M1 interradicular septum, suggesting that W9 does not have a strong osteogenic effect, but rather has anti-bone resorbing effects. In fact, administration of W9 to mice with calvarial defects promoted bone formation in the presence of BMP-2, but not in the absence of BMP-2 [33]. Furthermore, W9 protected ovariectomized- and low calcium diet- induced bone loss by suppressing bone resorption [28]. These previous and the present studies suggest that, unlike risedronate, W9 does not inhibit bone formation with the suppression of bone resorption. Therefore, it is likely that administration of W9 does not induce ectopic bone formation including osteophytes. Further studies are needed to clarify the effects of W9 on ectopic bone formation.

The most important clinical feature of periodontal disease is loss of alveolar bone. It is an urgent need in periodontal disease treatment to prevent alveolar bone loss. We have reported that *OPG*^–/–^mouse exhibited a marked alveolar bone loss without infections [11]. The distance between CEJ and ABC in *OPG*^*–/–*^mice was increased with time. Administration of risedronate and anti-RANKL antibody to *OPG*^*–/–*^mice prevented alveolar bone loss and suppressed the increase in the CEJ-ABC distance. These results suggest that antiresorptive agents can be therapeutic agents for periodontitis. However, it is well known that antiresorptive agents induce osteonecrosis of the jaw in patients with cancer [38, 39]. Antiresorptive agents, such as bisphosphonates and anti-RANKL antibody, strongly inhibit bone formation as well as bone resorption [9, 17–20, 24]. Inhibition of bone formation by these agents may be related to the development of osteonecrosis of the jaw. W9 inhibited bone resorption, but still stimulated bone formation to prevent alveolar bone loss in *OPG*^*–/–*^mice. Thus, the mechanism of W9 in suppressing alveolar bone loss is different from that of antiresorptive agents. This suggests that W9 can become a new therapeutic drug for osteoporosis. Indeed, parathyroid hormone, a bone formation agent in osteoporosis, was reported to protect alveolar bone loss in animal and human studies [40, 41]. Further studies are need to validate effects of W9 on alveolar bone loss in periodontitis models.

In addition, W9 may exert some effects on inflammation in periodontitis models. Previous studies have shown that the effect of W9 on bone formation was not stimulated by knockdown of TNF-α and deficiency of TNF receptor type I [29, 30]. TNF-α-induced inflammation signals are believed to be important signals for development of periodontal disease [42]. However, effects of W9 on inflammations are important points for applying W9 to treatments of periodontal disease.

In general, peptides have a short biological half-life due to their low stability in systemic circulation [43]. Therefore, repetitive administration and larger dosage of peptides are needed to obtain their effects. In fact, previous studies reported that the repetitive administration of W9 with high doses increased bone mass [28, 44]. In contrast, bisphosphonates including risedronate are effective at small doses because they accumulated in bone matrices without degradation [45].

Furuya *et al*. [29] showed that W9 administration for 5 days increased cortical bone formation in mice. We also showed that the short-term (for 5 days) treatment with W9 inhibited bone resorption and promoted bone formation in alveolar bone of *OPG*^–/–^mice. Effects of long-term treatment with W9 on alveolar bone and other tissues have not been evaluated in the present study. The previous studies [28, 44] reported that long-term treatments with W9 increased bone mass and promoted bone maturation without any adverse effect. Therefore, the long-term treatment can be expected to further increase alveolar bone in *OPG*^–/–^mice.

In conclusion, we demonstrated that administration of W9 effectively prevented alveolar bone loss caused by OPG deficiency. We have proposed the possibility that beneficial effects of W9 on bone are caused by two different mechanisms: suppression of RANK-mediated signals in osteoclast precursors and enhancement of RANKL–mediated signals in osteocytes. Thus, the effects of W9 on bone are quite unique. W9 may become a unique drug for treatment of patients with osteoporosis and periodontitis. Further experiments using other bone loss models, such as ovariectomy and rheumatoid arthritis, are necessary for the development of W9 as a drug for bone health.

## Supporting information

S1 Fig Effects of W9 on survival of osteoclasts supported by RANKL or IL-1α. Osteoblasts obtained from mouse calvariae and bone marrow cells were co-cultured in the presence of 1,25-dihydroxyvitamin D_3_ (10^−8^ M) and prostaglandin E_2_ (10^−6^ M) in 100-mm diameter dishes pre-coated with collagen gels (Nitta Gelatin, Osaka, Japan) [S1 Text, 1]. Osteoclasts formed on day 7 were released from the dishes by treatment with 0.2% collagenase (Wako Pure Chemical, Osaka). The crude osteoclast preparation was replaced on 24-well culture plates for 5-hour culture. The plates were treated with with trypsin-EDTA to remove osteoblastic cells [S1 Text, 2]. Most of the remaining cells were multinucleated osteoclasts (purified osteoclast preparation). (A) Purified osteoclasts were further cultured in the presence or absence of RANKL (200 ng/ml) or IL-1α (2.5 ng/ml). W9 (100 μM) was added to some cultures. After culture for 40 hours, cells were stained for TRAP. (B) The number of TRAP-positive osteoclasts was counted before and after culture for 40 hours. The percentages of the surviving osteoclasts were calculated. Data are expressed as the means ± SD. *: p<0.05. Purified osteoclasts spontaneously died via apoptosis, and both RANKL and IL-1α promoted the survival of osteoclasts. The survival of osteoclasts supported by RANKL but not by IL-1α was suppressed by W9.
(TIF)

S2 Fig Effects of W9 on ALP activity and mineralization of primary osteoblasts in *OPG*^*-/-*^ mice. Primary osteoblasts were prepared from newborn mouse calvariae from WT and *OPG*^–/–^mice [S1 Text, 3]. (A) Osteoblasts (1 x 10^5^ cells/well) were cultured in the presence of 100 μg/ml ascorbic acid and 5 mM β-glycerophosphate (Wako) in αMEM (Sigma, St. Louis, MO) containing 10% fetal bovine serum (FBS) (JRH Biosciences, Lenexa, KS) in 6-well collagen-coated plates (osteogenic culture conditions). The cultures were treated with or without increasing concentrations of W9. After culture for 14 days, cells were processed for alkaline phosphatase (ALP) staining. After culture for 21 days, cells were processed for alizarin red staining as described previously [S1 Text, 4].
(TIF)

S3 Fig Effects of W9 and risedronate administration on alveolar bone in WT mice. W9 or risedronate was injected into 12-week-old WT mice. On day 6, bone volume/tissue volume (BV/TV) of the first molar (M1) interradicular septum, a part of alveolar bone, was measured by μCT images. (A) μCT images of the interradicular septum of the M1 in mandibles from WT mice (an area surrounded by a white dotted line). (B) BV/TV was measured in the interradicular M1 septum from WT mice treated with and without W9 or risedronate (*n* = 5). Data are expressed as the mean ± SD in (B). Scale bar, 0.5 mm.
(TIF)

S1 Text Supporting information references list. (DOCX)
