# Supplementary figures and images for: Treatment of OPG-deficient mice with WP9QY, a RANKL-binding peptide, recovers alveolar bone loss by suppressing osteoclastogenesis and enhancing osteoblastogenesis
Source: PLoS One. 2017 Sep 22;12(9):e0184904. doi: 10.1371/journal.pone.0184904 (PMC5609750; doi:10.1371/journal.pone.0184904)

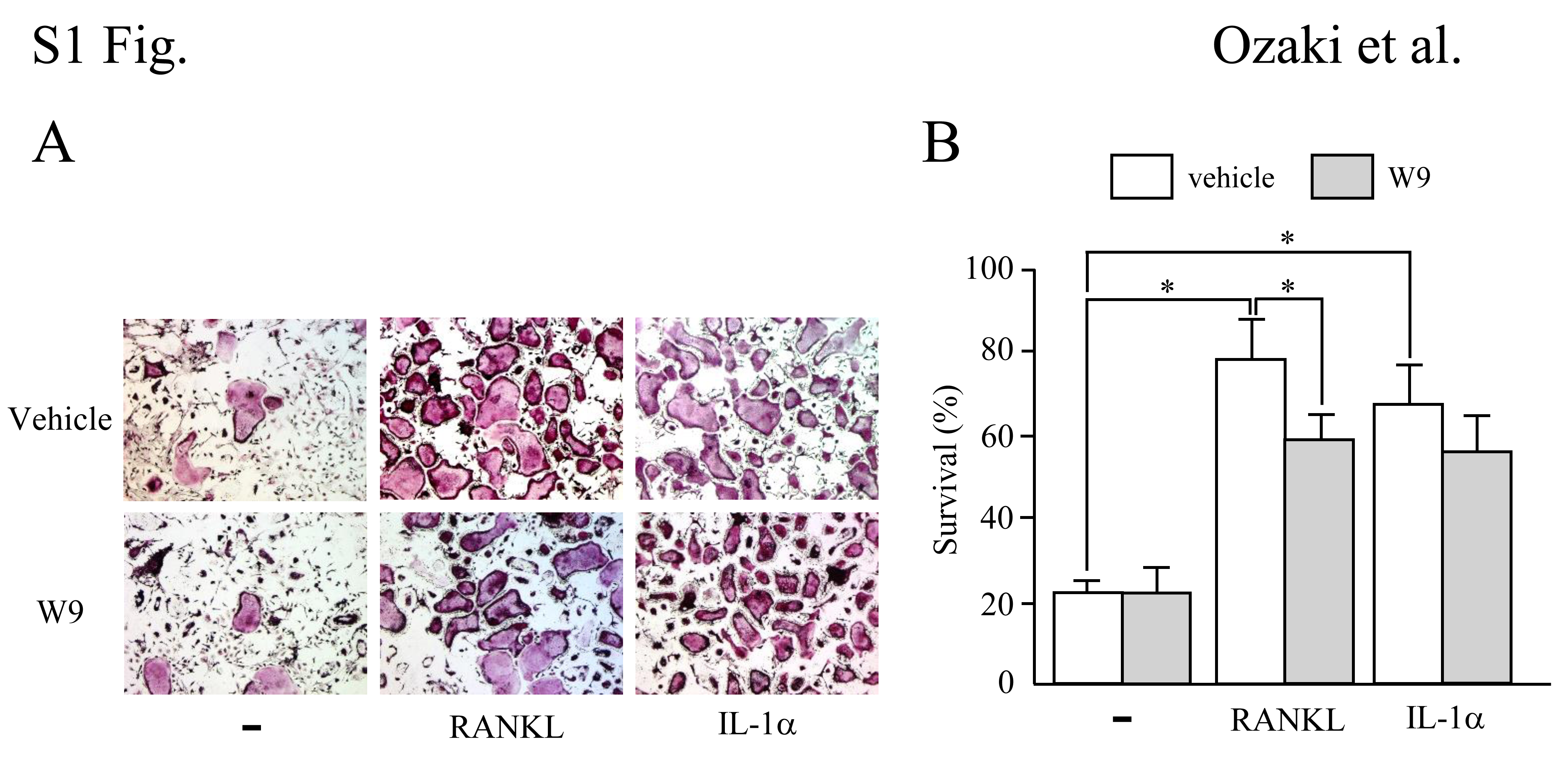

Supplement: S1 Fig — Osteoblasts obtained from mouse calvariae and bone marrow cells were co-cultured in the presence of 1,25-dihydroxyvitamin D3 (10−8 M) and prostaglandin E2 (10−6 M) in 100-mm diameter dishes pre-coated with collagen gels (Nitta Gelatin, Osaka, Japan) [S1 Text, 1]. Osteoclasts formed on day 7 were released from the dishes by treatment with 0.2% collagenase (Wako Pure Chemical, Osaka). The crude osteoclast preparation was replaced on 24-well culture plates for 5-hour culture. The plates were treated with with trypsin-EDTA to remove osteoblastic cells [S1 Text, 2]. Most of the remaining cells were multinucleated osteoclasts (purified osteoclast preparation). (A) Purified osteoclasts were further cultured in the presence or absence of RANKL (200 ng/ml) or IL-1α (2.5 ng/ml). W9 (100 μM) was added to some cultures. After culture for 40 hours, cells were stained for TRAP. (B) The number of TRAP-positive osteoclasts was counted before and after culture for 40 hours. The percentages of the surviving osteoclasts were calculated. Data are expressed as the means ± SD. *: p<0.05. Purified osteoclasts spontaneously died via apoptosis, and both RANKL and IL-1α promoted the survival of osteoclasts. The survival of osteoclasts supported by RANKL but not by IL-1α was suppressed by W9. (TIF) [file pone.0184904.s001.tif]

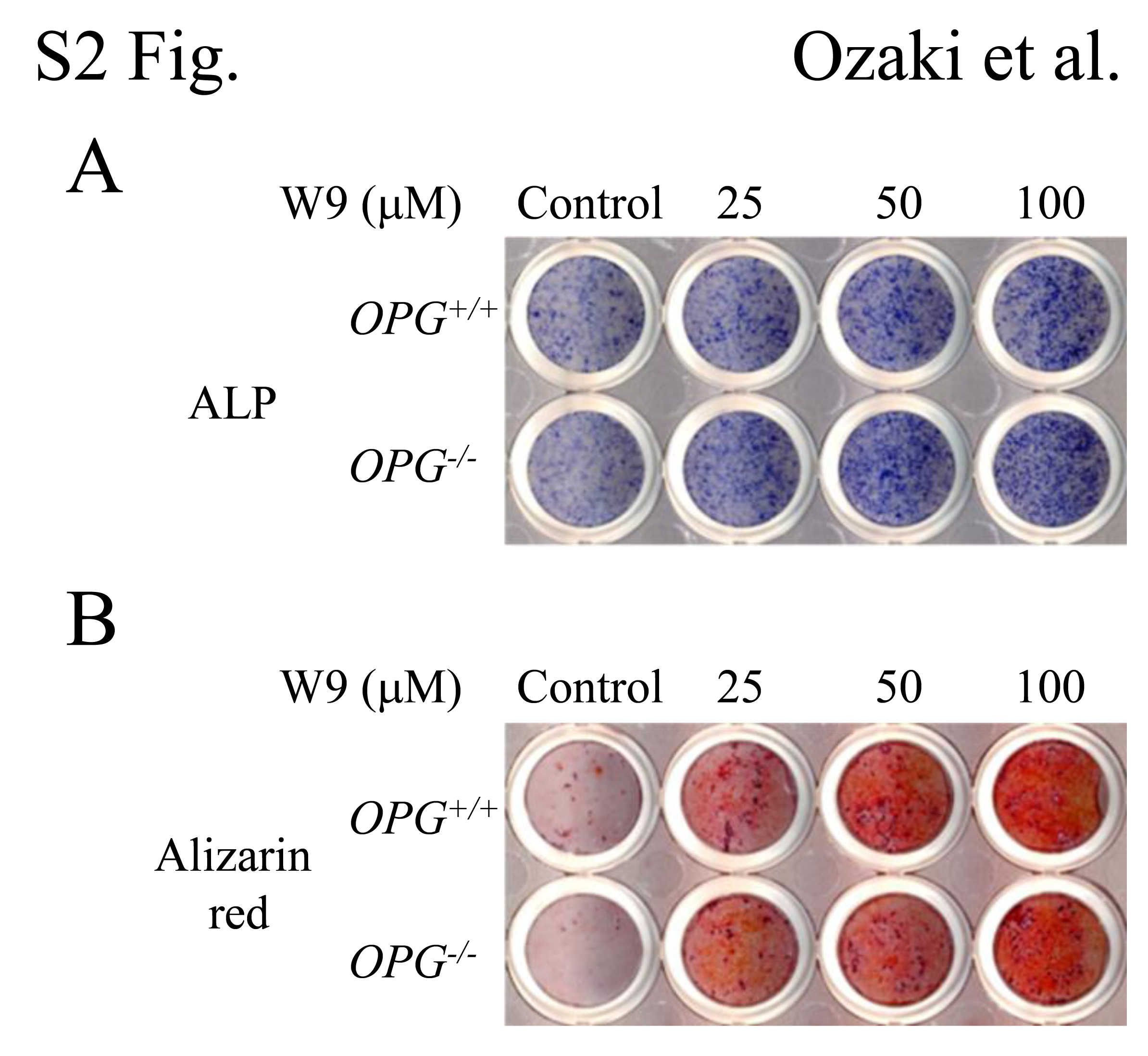

Supplement: S2 Fig — Primary osteoblasts were prepared from newborn mouse calvariae from WT and OPG–/–mice [S1 Text, 3]. (A) Osteoblasts (1 x 10^5^ cells/well) were cultured in the presence of 100 μg/ml ascorbic acid and 5 mM β-glycerophosphate (Wako) in αMEM (Sigma, St. Louis, MO) containing 10% fetal bovine serum (FBS) (JRH Biosciences, Lenexa, KS) in 6-well collagen-coated plates (osteogenic culture conditions). The cultures were treated with or without increasing concentrations of W9. After culture for 14 days, cells were processed for alkaline phosphatase (ALP) staining. After culture for 21 days, cells were processed for alizarin red staining as described previously [S1 Text, 4]. (TIF) [file pone.0184904.s002.tif]

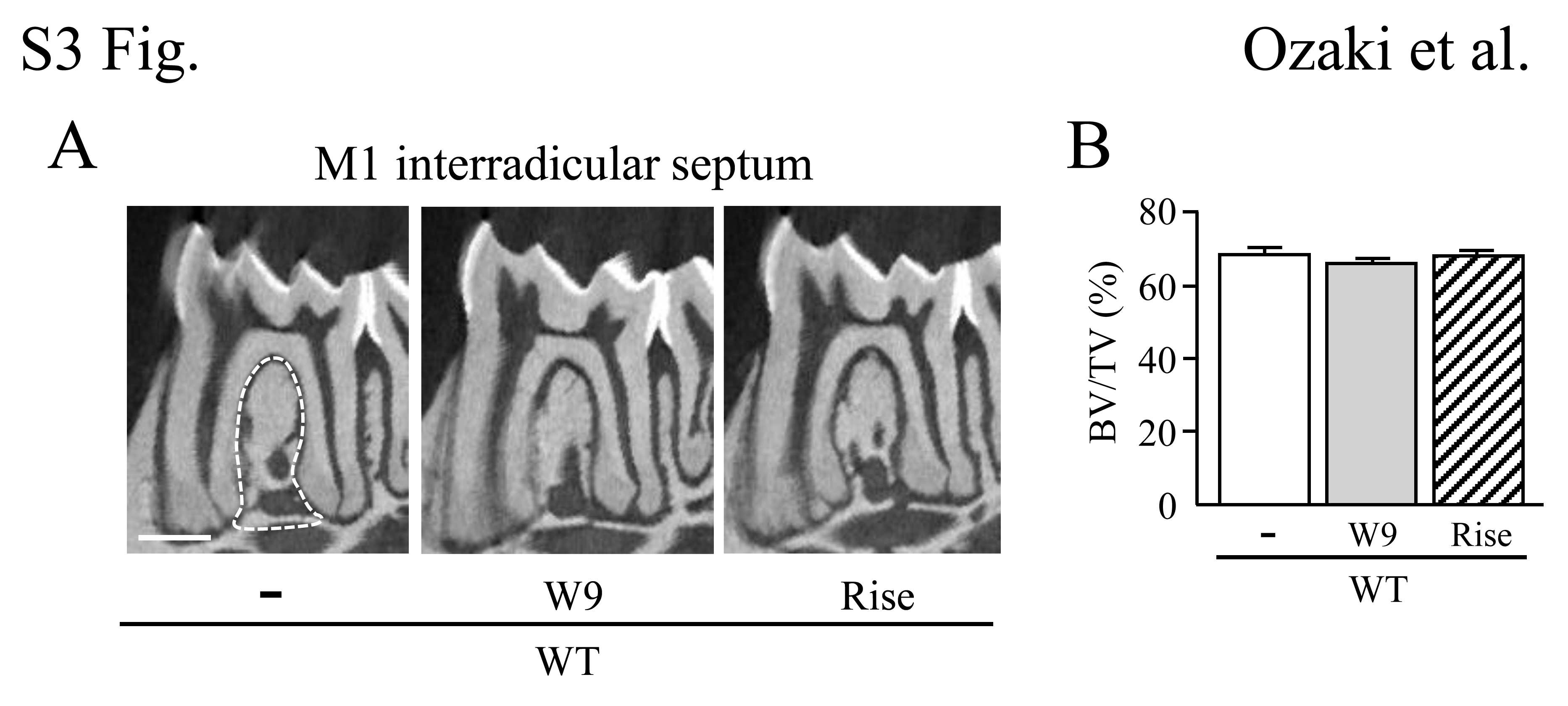

Supplement: S3 Fig — W9 or risedronate was injected into 12-week-old WT mice. On day 6, bone volume/tissue volume (BV/TV) of the first molar (M1) interradicular septum, a part of alveolar bone, was measured by μCT images. (A) μCT images of the interradicular septum of the M1 in mandibles from WT mice (an area surrounded by a white dotted line). (B) BV/TV was measured in the interradicular M1 septum from WT mice treated with and without W9 or risedronate (n = 5). Data are expressed as the mean ± SD in (B). Scale bar, 0.5 mm. (TIF) [file pone.0184904.s003.tif]
