# Supplementary material for: Treatment of OPG-deficient mice with WP9QY, a RANKL-binding peptide, recovers alveolar bone loss by suppressing osteoclastogenesis and enhancing osteoblastogenesis
Source: PLoS One. 2017 Sep 22;12(9):e0184904. doi: 10.1371/journal.pone.0184904 (PMC5609750; doi:10.1371/journal.pone.0184904)
Supplement: S1 Text — (DOCX) [file pone.0184904.s004.docx]

**Supporting information references**

1. Takahashi N, Akatsu T, Udagawa N, Sasaki T, Yamaguchi A, Moseley JM, et al. Osteoblastic cells are involved in osteoclast formation. Endocrinology. 1988; 123(5):2600-2. doi: 10.1210/endo-123-5-2600. PMID: 2844518.
2. Jimi E, Nakamura I, Ikebe T, Akiyama S, Takahashi N, Suda T. Activation of NF-kappaB is involved in the survival of osteoclasts promoted by interleukin-1. J Biol Chem. 1998; 273(15):8799-805. PMID: 9535858.
3. Kinugawa S, Koide M, Kobayashi Y, Mizoguchi T, Ninomiya T, Muto A, et al. Tetracyclines convert the osteoclastic-differentiation pathway of progenitor cells to produce dendritic cell-like cells. J Immunol. 2012; 188(4):1772-81. doi: 10.4049/jimmunol.1101174. PMID: 22250082.
4. Okamoto M, Udagawa N, Uehara S, Maeda K, Yamashita T, Nakamichi Y, et al. Noncanonical Wnt5a enhances Wnt/beta-catenin signaling during osteoblastogenesis. Sci Rep. 2014; 4:4493. doi: 10.1038/srep04493. PMID: 24670389; PubMed Central PMCID: PMCPMC3967152.
